# Parametric study of hydrogenic inventory in the ITER divertor based on machine learning

**DOI:** 10.1038/s41598-020-74844-w

**Published:** 2020-10-20

**Authors:** Rémi Delaporte-Mathurin, Etienne Hodille, Jonathan Mougenot, Gregory De Temmerman, Yann Charles, Christian Grisolia

**Affiliations:** 1 CEA, IRFM, 13108 Saint-Paul-lez-Durance, France; 2 Laboratoire des Sciences des Procédés et des Matériaux, LSPM, CNRS, UPR 3407, Université Sorbonne Paris Nord, 93430 Villetaneuse, France; 3 ITER Organization, Route de Vinon sur Verdon, CS 90 046, 13067 Saint-Paul-lez-Durance Cedex, France

**Keywords:** Nuclear fusion and fission, Applied mathematics, Energy science and technology, Nuclear energy

## Abstract

A parametric study is performed with the 2D FESTIM code for the ITER monoblock geometry. The influence of the monoblock surface temperature, the incident ion energy and particle flux on the monoblock hydrogen inventory is investigated. The simulated data is analysed with a Gaussian regression process and an inventory map as a function of ion energy and incident flux is given. Using this inventory map, the hydrogen inventory in the divertor is easily derived for any type of scenario. Here, the case of a detached ITER scenario with inputs from the SOLPS code is presented. For this scenario, the hydrogen inventory per monoblock is highly dependent of surface temperature and ranges from $10^{18}$ to $6 \times 10^{19}$ H after a $10^{7}$ s exposure. The inventory evolves as a power law of time and is lower at strike points where the surface temperature is high. Hydrogen inventory in the whole divertor after a $10^{7}$ s exposure is estimated at approximately 8 g.

## Introduction

The understanding of hydrogen behaviour in materials of fusion devices is crucial for several reasons. First the ITER in-vessel safety limit of tritium inventory of $1\,\hbox {kg}$ must be ensured in order to limit the risk of release in case of an accident^1^. Hydrogen in materials can also lead to embrittlement phenomenon which could eventually decrease components’ lifetime^2^. In addition, outgassing from material during long plasma discharges can cause uncontrolled increase of the core plasma density^3^.

In tokamaks, the highest particle fluxes are located on the divertor. The ITER divertor is composed of 54 cassettes. Each one of them is made of plasma-facing components (namely the inner and outer vertical targets and the dome)^4^. These are composed of monoblocks and are exposed to intense particle and heat loads. A first estimate of hydrogen retention in monoblocks was made in^5^ by performing multidimensional hydrogen transport simulations with FESTIM. It was shown that when hydrogen concentration and/or thermal fields are 2D, the 1D approximation is not sufficient to fully understand the behaviour of plasma facing components in tokamaks. The particle and heat flux used in these simulations were however too high to be representative of the whole divertor and simulating the whole divertor domain remains a major challenge.

Decreasing the particle flux at the surface of the monoblock will tend to decrease the mobile particles concentration which would decrease the trap occupancy. However, it will also decrease the heat load on the surface of the monoblock leading to a decrease of the surface temperature and an increase of the trap occupancy. Moreover, this will tend to reduce the amount of implanted particle which will desorb at the surface. There is therefore a trade-off between the implanted particle flux and the monoblock surface temperature.

The first goal of this study was to estimate the total hydrogen inventory in ITER divertor. To this end, FESTIM simulations of ITER-like monoblocks were performed. Instead of simulating each monoblock of the divertor individually (which would be computatively expensive), a parametric study was made to obtain a mapping of the component’s response to several parameters such as incident particle flux, ion energy, heat flux and surface temperature. This large volume of simulated data (several hundred simulations) was then analysed in order to extract knowledge from it. Machine learning algorithms were used to map the global solution onto a continuous parameter space so that one can easily test several exposure conditions without having to run all the simulations again but simple projections onto the parameters space. Finally, the results were applied to the expected ITER divertor conditions representative of a detached plasma scenario calculated by SOLPS^6^ in order to estimate the hydrogen inventory in the whole divertor.

## Methodology

### Model description

As described in previous studies^5,7–9^, the macroscopic rate equations model used in this work splits hydrogen isotopes into two populations: the mobile particles and the trapped ones. The temporal evolution of mobile particles $c_{\mathrm {m}}$ and trapped particles $c_{\mathrm {t,i}}$ in the i-th trap are described in Eqs. (1) and (2) respectively.

1 $$\begin{aligned}&\frac{\partial c_{\mathrm {m}}}{\partial t}=\vec {\nabla } \cdot \left( D(T) \vec {\nabla }c_{\mathrm {m}}\right) +S-\sum \frac{\partial c_{\mathrm {t}, i}}{\partial t} \end{aligned}$$

2 $$\begin{aligned}&\frac{\partial c_{\mathrm {t}, i}}{\partial t}=k(T) \cdot c_{\mathrm {m}} \cdot \left( n_{i}-c_{\mathrm {t}, i}\right) -p(T) \cdot c_{\mathrm {t}, i} \end{aligned}$$

In Eq. (1), ${D(T)=D_0 \cdot \exp \big (-E_{\mathrm {diff}}/ (k_B \cdot T )\big )}$ is the diffusion coefficient in $\hbox {m}^{2}\,\hbox {s}^{-1}$, *T* the temperature in $\hbox {K}$ and ${k_B = 8.617 \times 10^{-5} \hbox {eV}\,\hbox {K}^{-1}}$ the Boltzmann constant, *S* is the volumetric source term of mobile particles in $\hbox {m}^{-3}\hbox {s}^{-1}$ (which can take into account plasma implantation), $k(T)=k_0\exp {\big (-E_{k} / (k_B \cdot T ) \big )}$ and $p(T)=p_0\exp {\big (-E_{p}/ (k_B \cdot T )\big )}$ are the trapping and detrapping rates expressed in $\hbox {m}^{3}\hbox {s}^{-1}$ and $\hbox {s}^{-1}$ respectively. $n_i$ is the trap density in $\hbox {m}^{-3}$. Concentration conservation is assumed at interfaces for the sake of simplicity and computation time. It has already been shown that retention in monoblocks is dominated by retention in tungsten^5^. Therefore, conservation of chemical potential will not have much influence on the results. A more complete description of this model is given in^5^.

The temperature temporal evolution is governed by the heat equation described as follow:

3 $$\begin{aligned} \rho \cdot C_p \frac{\partial T}{\partial t}=\vec {\nabla } \cdot (\lambda \vec {\nabla } T) \end{aligned}$$

where $\rho$ is the density of the material in $\hbox {kg}\,\hbox {m}^{-3}$, $C_p$ its specific heat capacity expressed in $\hbox {J}\,\hbox {kg}^{-1}\,\hbox {K}^{-1}$ and $\lambda$ the thermal conductivity expressed in $\hbox {W K}^{-1}$.

Equations (1), (2) and (3) are then solved in FESTIM using the finite element method implemented in the FEniCS project^10^. FESTIM is implemented in Python and provides a user-friendly interface for performing multiphysics, multidimensional and multi-material simulations^5^. All plots in this work were generated with Matplotlib^11^.

### Simulation description

The first step of the work was to simulate the hydrogenic transport and trapping in a tungsten monoblock as a function of the loading conditions.

Moreover, a parametric study will be carried out in order to simulate the whole range of the implantation conditions encountered in the ITER divertor.

#### Geometry

**Figure 1:**
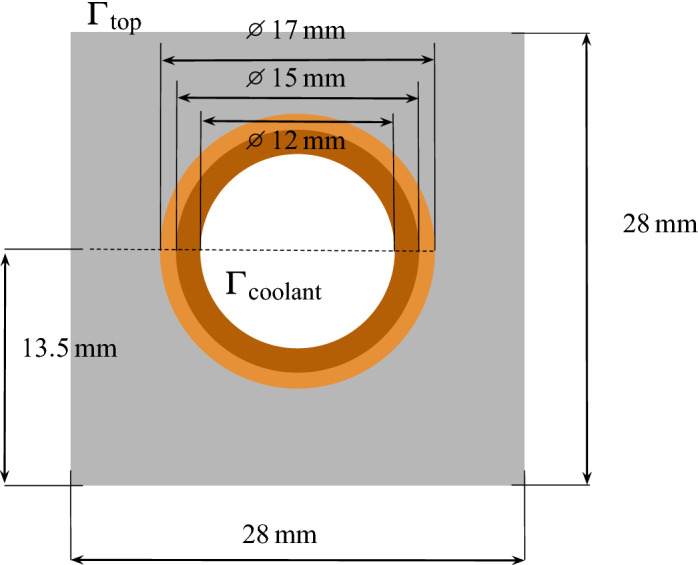
Monoblock geometry showing W armour  , Cu interlayer  , CuCrZr alloy cooling pipe  .

The geometry used in this work is that of a non-shaped ITER monoblock (see Fig. 1). The monoblocks use tungsten armour and a $1.5\,\hbox {mm}$-thick CuCrZr pipe as heat sink. The pipe is jointed to the tungsten. A 1 mm-thick Cu interlayer is used in order to handle stress resulting from differential thermal expansion^12^. The surface $\Gamma _{{\mathrm {top}}}$ is facing the plasma and $\Gamma _{\mathrm {coolant}}$ is cooled by water.

#### Material properties

**Figure 2:**
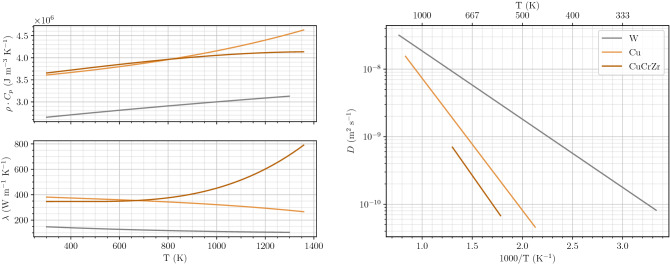
Material properties used in the simulations^13–15^.

The material properties used in these simulations are described in Table 1 and their temperature dependence is shown in Fig. 2. The trap parameters are described in Table 2. Influence of mechanical fields such as thermal expansion on trap creation^7^ was not taken into account in this work. Hodille et al. described an extrinsic trap in tungsten created by ion implantation^9^. This trap is assumed to have only a small influence on the macroscopic behaviour of the monoblock and is therefore not taken into account in this work for the sake of simplicity.

**Table 1:** Materials properties used in the simulations.

Material	Thermal properties		Hydrogen transport	
	$\rho \cdot C_p (\hbox {J}\,\hbox {K}^{-1}\,\hbox {m}^{-3})$	$\lambda ({\hbox {W}\,\hbox {K}^{-1}})$	$D_0\,(\hbox {m}^2\,\hbox {s}^{-1})$	$E_{\mathrm {diff}}(\hbox {eV})$
W	$5.1\times 10^{-6} \cdot T^3 - 8.3\times 10^{-2}\cdot T^2 + 6.0 \times 10^{2}\cdot T +2.4\times 10^6$	$-7.8\times 10^{-9}\cdot T^3 +5.0\times 10^{-5}\cdot T^2 -1.1\times 10^{-1} \cdot T +1.8\times 10^{2}$	$1.9\times 10^{-7}$	0.20
Cu	$1.7\times 10^{-4}\cdot T^3 +6.1\times 10^{-2}\cdot T^2 +4.7\times 10^2\cdot T +3.5\times 10^6$	$-3.9\times 10^{-8}\cdot T^3 +3.8\times 10^{-5}\cdot T^2 -7.9\times 10^{-2}\cdot T +4.0\times 10^2$	$6.6\times 10^{-7}$	0.39
CuCrZr	$-1.8\times 10^{-4}\cdot T^3 +1.5\times 10^{-1}\cdot T^2 +6.2\times 10^2\cdot T +3.5\times 10^6$	$5.3\times 10^{-7}\cdot T^3 -6.5\times 10^{-4}\cdot T^2 +2.6\times 10^{-1}\cdot T +3.1\times 10^2$	$3.9\times 10^{-7}$	0.42

Thermal properties are fitted from ANSYS^13–15^.

**Table 2:** Traps properties used in the simulations^9,16^.

	Material	$k_0 ({\hbox {m}^3\,\hbox {s}^{-1}})$	$E_k (\hbox {eV})$	$p_0 ({\hbox {s}^{-1}})$	$E_p (\hbox {eV})$	$n_i (\hbox {at fr })$
Trap 1	W	$3.1 \times 10^{-16}$	0.20	$8.4 \times 10^{12}$	1.00	$1.1 \times 10^{-3}$
Trap 2	Cu	$6.0 \times 10^{-17}$	0.39	$8.0 \times 10^{13}$	0.50	$5.0 \times 10^{-5}$
Trap 3	CuCrZr	$1.2 \times 10^{-16}$	0.42	$8.0 \times 10^{13}$	0.85	$5.0 \times 10^{-5}$

#### Boundary conditions

Mobile particles concentration $c_{\mathrm {m}}$ is imposed on $\Gamma _{{\mathrm {top}}}$ which allows to simulate particle implantation without having to include a volumetric source term applied on the first few nanometres. This approximation allows to have a broader mesh and therefore saves computation time without affecting the macroscopic behaviour. Molecular recombination is assumed on $\Gamma _{\mathrm {coolant}}$. Even though it could be assumed on the gaps between monoblocks, it can be shown that its influence on the macroscopic behaviour remains low. Desorption from the other surfaces is therefore assumed to be zero for simplification purposes. Uniform heat loads $\varphi _H$ are applied on the surface $\Gamma _{{\mathrm {top}}}$ with a Neumman boundary condition or temperature is constrained on $\Gamma _{\mathrm {top}}$ with a Dirichlet boundary condition and a convective exchange condition is set on surface $\Gamma _{\mathrm {coolant}}$. All the other surfaces are assumed thermally insulated. The set of boundary conditions can finally be described as follow:

4 $$\begin{aligned} -\lambda \vec {\nabla } T \cdot \vec {n}&=\varphi _{H} \quad \text {or} \quad T = T_{\mathrm {surface}}\quad&\text{ on } \Gamma _{\mathrm {top}}\end{aligned}$$

5 $$\begin{aligned} \qquad \quad c_{\mathrm {m}}&= c_{\mathrm {surface}}\quad&\text{ on } \Gamma _{\mathrm {top}}\end{aligned}$$

6 $$\begin{aligned} -\lambda \vec {\nabla } T\cdot \vec {n}&= -h \cdot \left( T_{\mathrm {coolant}} - T\right) \quad&\text{ on } \Gamma _{\mathrm {coolant}}\end{aligned}$$

7 $$\begin{aligned} -D \vec {\nabla } c_{\mathrm {m}} \cdot \vec {n}&= K_{\mathrm {CuCrZr}} \cdot c_{\mathrm {m}}^{2} \quad&\text{ on } \Gamma _{\mathrm {coolant}} \end{aligned}$$

with $h=70,000\,\hbox {W}\,\hbox {m}^{-2}\,\hbox {K}^{-1}$ being the heat exchange coefficient calculated from the Sieder-Tate correlation for the forced convection regime, $T_{\mathrm {coolant}}= 323\,\hbox {K}$ and $\vec {n}$ the normal vector and $K_{\mathrm {CuCrZr}} = 2.9 \times 10^{-14}\cdot \exp {(-1.92/(k_B\cdot T))}$ the recombination coefficient of the copper alloy (in vacuum) expressed in $\hbox {m}^4\,\hbox {s}^{-1}$^17^.

## Results

### Thermal behaviour

**Figure 3:**
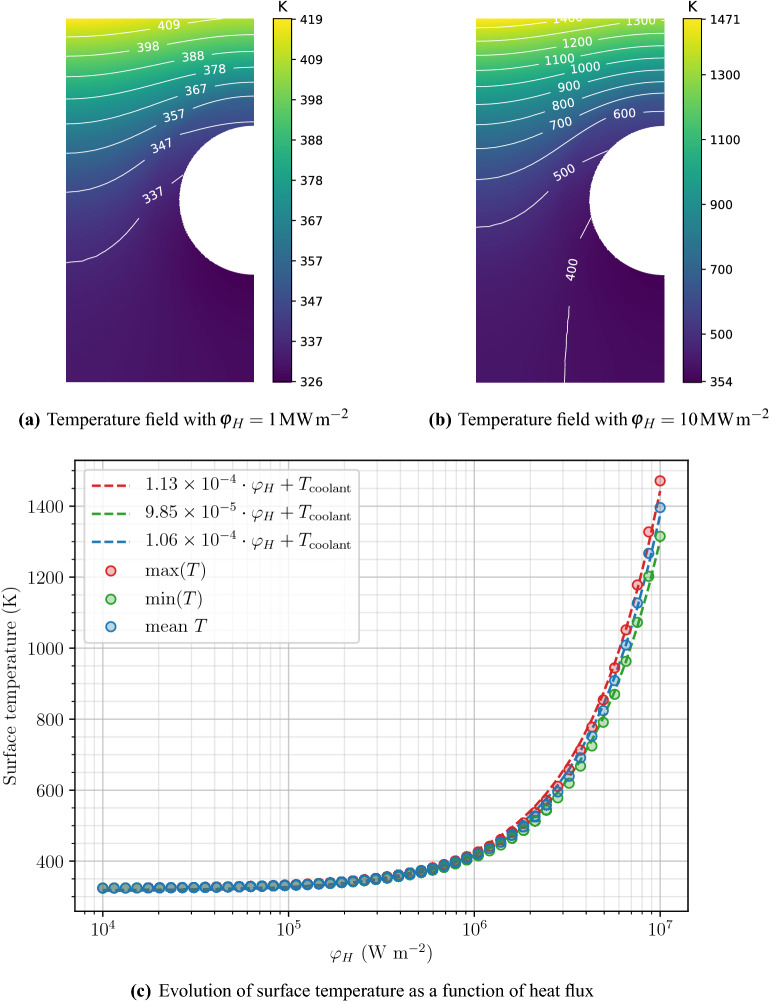
Thermal behaviour of the monoblock.

Steady-state heat transfer simulations were performed with FESTIM with varying heat loads $\varphi _H$. With $\varphi _H = 1\,\hbox {MW}\,\hbox {m}^{-2}$, the surface temperature of the monoblock was found to be around 400 K (see Fig. 3a) whereas with $\varphi _H = {10}\,\hbox {MW}\,\hbox {m}^{-2}$ the surface was around 1400 K (see Fig. 3b).

In order to simplify the analytical relations used in “Influence of incident particle flux and ion energy on hydrogen inventory” section, only the mean surface temperature was considered in the following sections. $T_{\mathrm {surface}}$ therefore increases linearly with the heat load and can be modelled by Eq. (8) (see Fig. 3c).

8 $$\begin{aligned} T_{\mathrm {surface}} = 1.1 \times 10^{-4} \cdot \varphi _H + T_{\mathrm {coolant}} \end{aligned}$$

This was found to be in very good agreement with experimental measurements performed in^18^.

### Influence of $T_{\mathrm {surface}}$ and $c_{\mathrm {surface}}$ on hydrogen inventory

In this section, the total inventory of hydrogen in monoblocks has been calculated as a function of $T_{\mathrm {surface}}$ and $c_{\mathrm {surface}}$. Temperature and mobile concentration of hydrogen were imposed with Dirichlet boundary conditions on $\Gamma _{\mathrm {top}}$ with $T_{\mathrm {surface}}$ varying from $T_{\mathrm {coolant}}$ to 1200 K and $c_{\mathrm {surface}}$ varying arbitrarily from $10^{20}\,\hbox {m}^{-3}$ to $6\times 10^{22}\,{\hbox {m}^{-3}}$. The assumption of a constant surface temperature had low influence on the results compared to a non-homogeneous surface temperature that could be obtained with a heat flux condition since surface temperature gradient was low compared to the one between the top surface and the cooling surface. For surface temperatures below 500  K, 1D simulations were performed for the penetration depth of hydrogen remained very low (a few microns) and 1D approximation was sufficient^19^. For temperatures above 500 K for which edge effects become dominant, 2D simulations have been performed.

**Figure 4:**
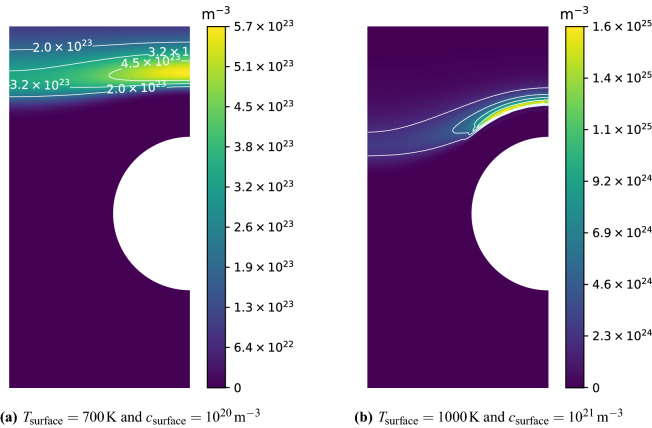
Retention fields in $\hbox {m}^{-3}$ after a $10^{7}\,\hbox {s}$ exposure.

After $10^{7}\,\hbox {s}$ a high retention zone appeared far from the exposed surface $\Gamma _{\mathrm {top}}$ (see Fig. 4). As described in^5^, this is due to thermal effects. As seen in Fig. 3a and b, the temperature was found to decrease in regions close to the cooling pipe $\Gamma _{\mathrm {coolant}}$ leading to an increase in trap occupancy, creating this high retention zone. This was however not true for monoblocks where $T_{\mathrm {surface}} \approx T_{\mathrm {coolant}}$ since the temperature gradient in the domain is very low. Instead, trap occupancy was close to one and the retention was high in the whole region where hydrogen had penetrated and not only far from the top surface.

**Figure 5:**
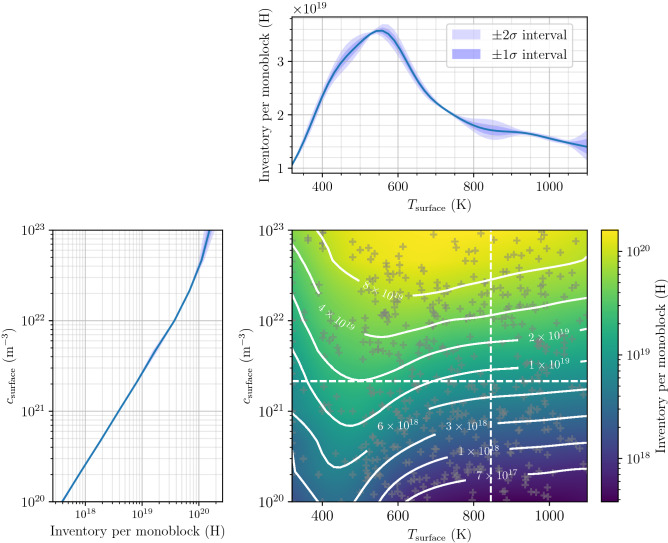
Evolution of the inventory after a $10^{7}\,\hbox {s}$ exposure as a function of $T_{\mathrm {surface}}$ and $c_{\mathrm {surface}}$ alongside with simulation points (grey crosses). The simulations points were fitted with a Gaussian regression process^22^ providing the standard deviation $\sigma$.

Hydrogen inventory in monoblocks as a function of $T_{\mathrm {surface}}$ and $c_{\mathrm {surface}}$ is shown in Fig. 5. In order to obtain this continuous field, more than 600 simulations randomly distributed on the parameter plane were run and analysed using a Gaussian process machine learning algorithm^20^ as in^21^ based on the python package inference-tools^22^. In Fig. 5, the inventory obtained by the Gaussian regression process is given for a constant value of $c_{\mathrm {surf}}={2\times 10^{21}}\,{\hbox {m}^{-3}}$ (top inset) and a constant temperature $T=850\,\hbox {K}$ (left inset). The Gaussian regression process was particularly appropriate as it calculates a confidence interval based on the standard deviation $\sigma$. As expected, the lower the density of simulation points, the higher was the value of $\sigma$ (for example around 850 K on the top inset of Fig. 5). However, despite the lack of simulation in this region, the value of $\sigma$ was still acceptable (only a few percents of the inventory) ensuring the quality of the resulting interpolation.

As expected, inventory was found to globally increase with $c_{\mathrm {surface}}$. For $T_{\mathrm {surface}} > 550\,\hbox {K}$, the inventory tended to decrease with surface temperature. However, for $T_{\mathrm {surface}} < 550\,\hbox {K}$, inventory increased with surface temperature. This phenomenon is due to a trade-off between an increase of the detrapping rate and an increase of the diffusion coefficient making the hydrogen particles penetrate deeper into the bulk. Above $550\,\hbox {K}$, detrapping becomes dominant and inventory decreases. This mapping of inventory as a function of $T_{\mathrm {surface}}$ and $c_{\mathrm {surface}}$ provides an easy way of estimating the inventory in monoblocks for several exposure conditions without having to run many simulations. Indeed, to estimate the inventory with different exposure conditions, one only needs to associate these conditions $(\varphi _{\mathrm {inc}}, E)$ to a couple $(c_{\mathrm {surf}}, T_{\mathrm {surf}})$.

### Influence of incident particle flux and ion energy on hydrogen inventory

Incident particle flux $\varphi _{\mathrm {inc}}$ and ion energy *E* have an impact on the amount of mobile particles implanted in the material but also on the heat load and therefore on the surface temperature of the monoblock.

Assuming a source term with a narrow Gaussian distribution and a non-instantaneous recombination (characterised by a recombination coefficient *K*), the concentration $c_{\mathrm {max}}$ at the near surface is approximated by:

9 $$\begin{aligned} c_{\mathrm {max}} = \frac{\varphi _{\mathrm {imp}} \cdot R_p}{D(T_{\mathrm {surface}})} + \sqrt{\frac{\varphi _{\mathrm {imp}}}{K(T_{\mathrm {surface}})}} \end{aligned}$$

where $\varphi _{\mathrm {imp}} = (1-r) \cdot \varphi _{\mathrm {inc}}$ is the implanted particle flux, *r* is the particle reflection coefficient, *K* is the recombination coefficient, and $R_p$ is the mean implantation depth in m. Details can be found in "appendix" as Supplementary Material. Many different values of the recombination coefficient *K* for tungsten can be found in literature. For instance the widely used Anderl coefficient describes an endothermic recombination^23^ whereas Ogorodnikova showed an exothermic recombination coefficient could be used to reproduce a set of experiments^24^.

Facing the difficulty of an accurate choice for *K* and following the recommendation of Causey et al.^25^, an instantaneous recombination will therefore be assumed (i.e. $K \rightarrow +\infty$). It is also worth noting that experiments by Bisson et al.^26^ support the fact that recombination is not the rate limiting step during the hydrogen release from polycrystalline tungsten after ion implantation.

In the following, the concentration on $\Gamma _{\mathrm {top}}$ was set to $c_{\mathrm {surface}} = c_{\mathrm {max}}$ for the kinetics involved are really fast (see appendix of^27^) and $R_p$ is small compared to the monoblock dimensions.

The heat load was assumed to evolve as a function of the incident particle flux $\varphi _{\mathrm {inc}}$ and *E* as follow:

10 $$\begin{aligned} \varphi _H = 2.2\cdot \varphi _{\mathrm {inc}} \cdot e \cdot (E + {13.6}\hbox {eV}) \end{aligned}$$

with $e = 1.6\,10^{-19}\hbox {C}$. This relation was obtained by fitting SOLPS data^28,29^. The factor 2.2 was applied to take into account other heat sources such as radiative flux.

Moreover, the ion energy *E* has an influence on *r* and implantation range $R_p$ and it was possible to model the evolution of these parameters with SRIM^30^ calculations as follow:

11 $$\begin{aligned} r= & \, 2\times 10^{-8} \cdot E^2 -6 \times 10^{-5} \cdot E + 8\times 10^{-1} \end{aligned}$$

12 $$\begin{aligned} R_p= & \, 1.4\times 10 ^{-10}\cdot E^{0.64} \end{aligned}$$

**Figure 6:**
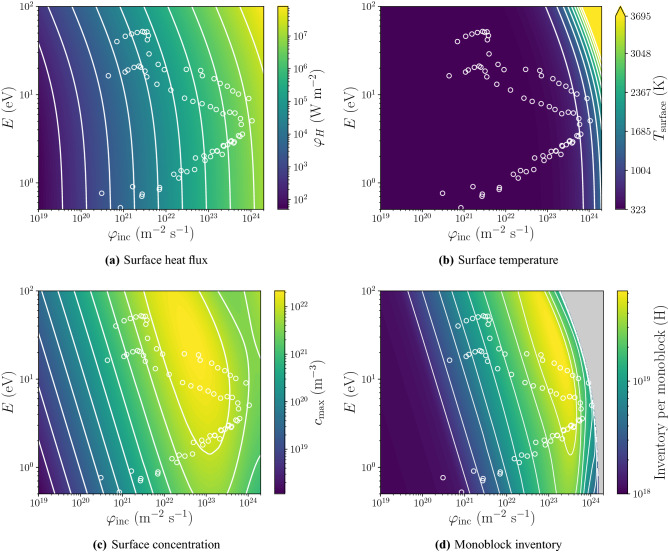
$\varphi _H$, $T_\text {surface}$, $c_{\mathrm {max}}$ and inventory per monoblock as a function of $\varphi _{\mathrm{inc}}$ and *E*. Inventory has not been calculated for surface temperature above 1200 K (greyed region). White circles correspond to points on ITER divertor using the divertor plasma parameters from SOLPS^6^ calculations (see “Application to tokamak exposure conditions” section).

By combining Eqs. (10), (11), one can obtain the evolution of $\varphi _H$ as a function of $\varphi _{\mathrm {inc}}$ and *E* as shown in Fig. 6a. From the thermal behaviour given by Eq. (8), the surface temperature $T_{\mathrm {surface}}$ can be computed (see Fig. 6b). Finally, $c_{\mathrm {max}}$ was obtained from Eqs. (9) and (12) (see Fig. 6c).

One must be aware that above 1500 K, W recrystallisation can occur and H transport will strongly be affected. The hypothesis made above as well as material properties may then not be valid. Because of the trade-off between the amount of implanted particles and the resulting heat flux, the maximum value of $c_{\mathrm {max}}$ was found to be $2\times 10^{22}\, \hbox {m}^{-3}$ around $(\varphi _{\mathrm {inc}}, E)=(8\times 10^{22}\hbox {m}^{-2}\,\hbox {s}^{-1}, 20\,\hbox {eV})$. Considering the previously calculated response of the monoblock to $c_{\mathrm {surface}}$ and $T_{\mathrm {surface}}$ (see Fig. 5), the inventory as a function of $\varphi _{\mathrm {inc}}$ and *E* was computed (see Fig. 6d). The inventory values have not been calculated for surface temperatures above 1200 K. Again a trade-off was found between implanted particle flux and surface temperature. Indeed, the maximum inventory was not found at regions where the incident flux is maximum but rather at regions where $c_{\mathrm {surface}}$ is maximum and $T_{\mathrm {surface}}$ is minimum as seen in previous 1D studies^8^.

### Application to tokamak exposure conditions

Each white circle in Fig. 6 corresponds to a point along a poloidal section of the ITER divertor for which implanted particle flux and ion energy were calculated with SOLPS^6^ for a partially detached plasma scenario. This scenario corresponds to a $Q=10$ discharge with a neutral pressure of 8.6 Pa^31^.

**Figure 7:**
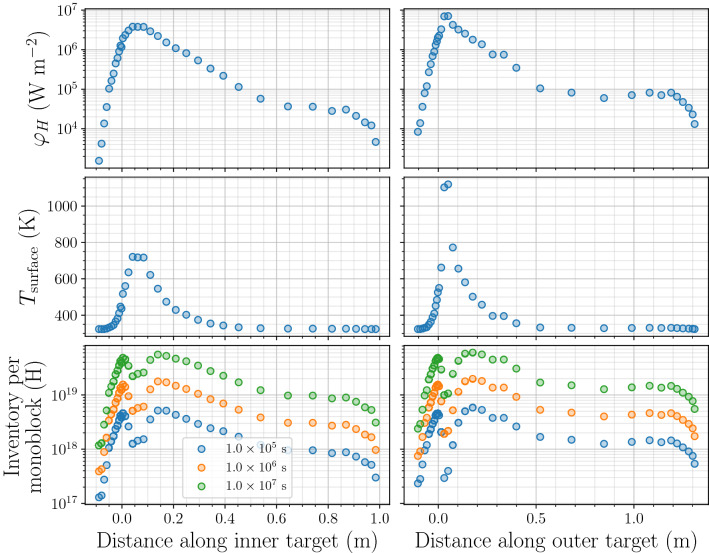
Evolution of $\varphi _H$ (top), $T_{\mathrm {surface}}$ (middle) and inventory (bottom) after several exposure times along a poloidal section of the divertor for inner vertical target and outer vertical target.

As expected the highest surface temperatures and heat loads were located on strike points and most of the zones on the divertor were found to stay at coolant temperature (see Fig. 7). The maximum hydrogen content is approximately $6\times 10^{19}\,\hbox {H}$ per monoblock after a $10^{7}\,\hbox {s}$ exposure. As explained in the previous section, the maximum inventory is not necessarily in the region where the flux is maximum as it induces a higher temperature which will tend to increase detrapping: strike points are not where hydrogen is trapped the most. Instead, the maximum inventory is reached about 5 cm away from the strike points where the temperature and the fluxes are high enough to guarantee a strong source of mobile particle but the temperature is not high enough to trigger detrapping.

**Figure 8:**
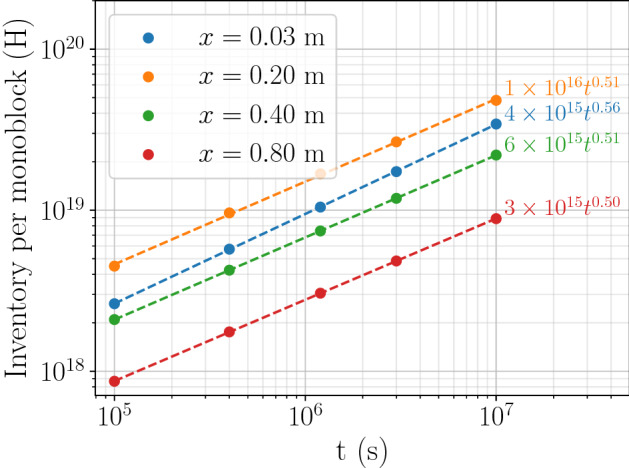
Temporal evolution of hydrogen inventory in monoblocks at several locations on inner vertical target of ITER divertor.

**Figure 9:**
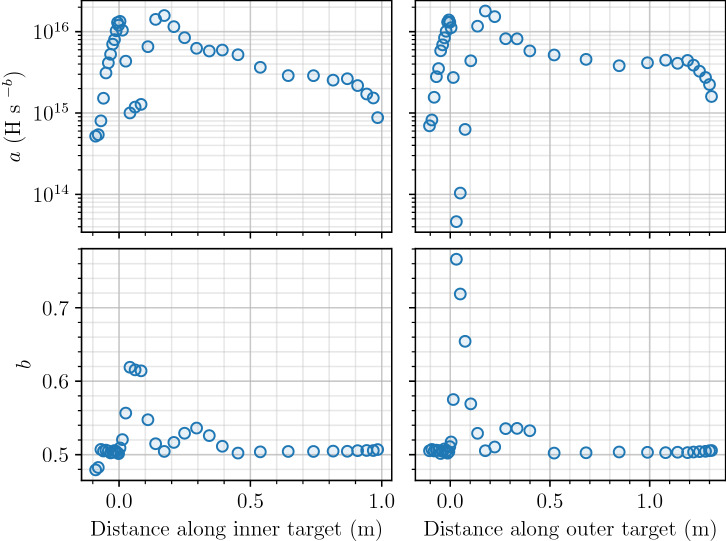
Evolution of coefficients *a* and *b* along a poloidal section of the divertor. Inventory evolves as $a\cdot t^b$ (H).

For all points on the divertor, the inventory evolved as $a \cdot t^b$ as shown in Fig. 8 for particular points on the inner vertical target ($x=0.03$ m is close to the strike point). The coefficient *b* is maximum on strike points reaching 0.75 (see Fig. 9). In other regions, *b* is closer to 0.5. This result can be explained by the non-homogeneous temperature field in monoblocks with high heat loads. For monoblocks with a high surface temperature, as hydrogen penetrates deeper into the bulk, the bulk temperature decreases (see Fig. 3b) leading to an increase of the trap occupancy^8^. The exponent *b* is therefore higher than 0.5. For monoblock where $T_{\mathrm {surface}} \approx T_{\mathrm {coolant}}$ on the other hand, the temperature is homogeneous in the whole domain and $b=0.5$. This corresponds to a diffusion-limited behaviour.

The temperature is close to $T_{\mathrm {coolant}}$ and the trap occupancy is therefore close to one in the whole domain which is not the case for regions near strike points where temperature fields are non-uniform.

One can obtain the inventory in the whole divertor by integrating the results obtained in Fig. 7 over the tokamak as follow:

13 $$\begin{aligned} {\mathrm {inv_{divertor}}} = N_{\mathrm {cassettes}} \cdot \left( N_{\mathrm {PFU-IVT}} \cdot \int {\mathrm {inv}}_{\mathrm{IVT}}(x)\, dx + N_{\mathrm {PFU-OVT}} \cdot \int {\mathrm {inv}}_{\mathrm {OVT}}(x) \, dx \right) \end{aligned}$$

with $N_{\mathrm {cassettes}}=54$ the number of cassettes, $N_{\mathrm {PFU-IVT}}=16$ and $N_{\mathrm {PFU-OVT}}=22$ the number of plasma facing units per cassette on the inner and outer targets respectively, ${\mathrm {inv}}_{\mathrm {IVT}}$ and ${\mathrm {inv}}_{\mathrm {OVT}}$ the hydrogen inventory profile along the inner and outer targets respectively and *x* the distance along the targets.

**Figure 10:**
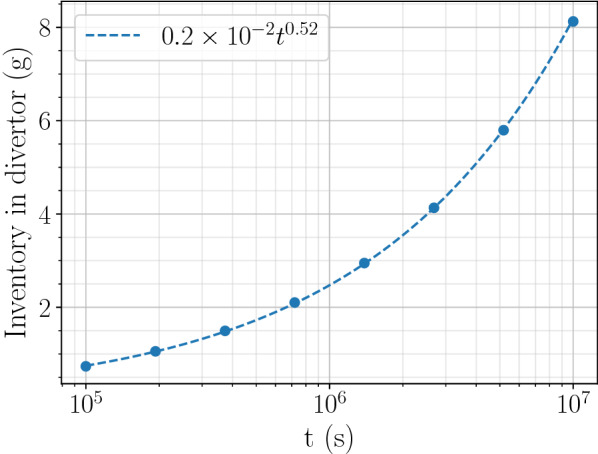
Temporal evolution of hydrogen inventory in the whole divertor.

After a $10^{7}\,\hbox {s}$ exposure, hydrogen inventory is estimated at approximately 8 g (see Fig. 10) which is relatively low considering the ITER in-vessel limit and the elapsed time. De Temmerman et al. showed that retention in ITER can reach 0.3 g per 400 s discharge when taking into account Be deposits.

### Discussion

If this methodology provides a rapid way of estimating hydrogen content in the whole divertor, several assumptions have however been made.

First, a steady state exposure was considered for simplification purposes. This result is however conservative. As seen in^5,8^, cycling effects could have an influence in regions where $T_{\mathrm {surface}}$ varies a lot, for example within 10 cm on both sides of the strike points. Though, since a large majority of monoblocks were found to stay at room temperature, even during operations (see Figs. 6b and 7) the thermal effect should remain low and discrepancies would rather be due to particle flux evolution along the target.

Shaping of monoblocks (e.g. chamfers) was not taken into account in this work for simplification purposes. Such shaping can have an influence on the incident particle and heat loads on the plasma facing surface of the monoblocks.

This study presents the hydrogen trapping in W monoblocks. It shows that the latter remains low but, as already pointed out by JET studies, the trapping on Be co-deposited layers is expected to be the main mechanism for tritium retention in ITER^32,33^. Such layers could be found in the cold regions of the divertor but as soon as the strike points hit these layers, they should be sputtered away (as sputtering of Be is possible even at low energy^32,34^). The retention where the deposited layers are not present (either sputtered or not formed anyway) would then be given by the model presented here.

The molecular recombination coefficient at the surface of the cooling pipe was taken from^17^ and was measured in vacuum. One could argue that recombination in presence of water will be facilitated. It can however be shown that this parameter has very low influence on the inventory since it was dominated by retention in tungsten. This parameter will however have an influence on the permeation flux and should be studied in future work.

Similarly, the influence on molecular recombination on the sides of the monoblock was found to have a low impact on the results. By assuming an instantaneous recombination coefficient, the relative error on the monoblock inventory was found to be significant only in hot regions (i.e. within 10 cm on both sides of the strike points). The influence on the total divertor inventory is therefore low (less than 5% after a $10^{7}\,\hbox {s}$ exposure) since it is dominated by regions where $T_{\mathrm {surface}} \approx T_{\mathrm {coolant}}$.

It should be noted that specific scenarii like edge localised modes (ELMs) were also not taken into account in this work since their time scale is very short. MRE simulations by Hu and Hassanein^35^ suggest that a 400 s discharge with 1 Hz or 10 Hz ELMs significantly reduces (77%) the inventory in W materials. However, the modelling of the ELM is simulated by increasing the temperature for a very short time without changing the incident flux of particles that can also be much higher thus balancing the fuel retention reduction. Another study by Schmid et al.^36^ also simulated the effect of 1 Hz ELMs on fuel retention in W. The outcome is that 6 s of 1 Hz-ELMs does not affect significantly the fuel retention, though the temperature excursion in those simulations are smaller than for the one of Hu and Hassanein. Thus, the effect of ELMs, especially the balance between increase of heat flux, incident energy and particle flux, could either favour or disfavour trapping, diffusion and migration and therefore the overall retention.

In this study the model to link the concentration of mobile particles at the surface (implantation zone) with the exposure condition considers that the particles are implanted in the bulk and that the recombination coefficient is very high since many uncertainties concerning the recombination coefficient are yet to be lifted. However, if an exothermic process is considered as in^24^, this should have low influence since recombination is very quick at a temperature close to that of the coolant.

On the other hand, experimental results^37^ suggest that for ion energy below 5 eV/H, typical of detached plasma as the one treated in the previous section, the surface process can be important and limits the uptake of hydrogen, i.e. the adsorption on the surface and the further absorption from surface to bulk could be the limiting process for the growth of $c_{\mathrm {surface}}$ during such exposure. The evolution of $c_{\mathrm {surface}}$ to the exposure condition for that range of energy would therefore be different and therefore the inventory. The advantage of the presented method is that taking into account such process is relatively easy as no expensive simulations are needed. One would only need to modify the model giving $c_{\mathrm {surface}}$ as a function of $(E_{\mathrm {inc}},\varphi _{\mathrm {inc}})$ to include the different surface processes. To this end, one can use kinetic surface models^38–41^.

Trap properties have a great impact on the inventory. In this study, a homogeneous trap distribution is assumed for simplification purposes. A more thorough study could investigate the influence on trap distribution, energy and density. Trap properties might also vary along the divertor based on exposure conditions. Moreover the impact of neutrons must be assessed as neutron-induced traps have a high detrapping energy.

Finally, helium implantation in the materials and bubble formation could modify the hydrogen transport in monoblocks.

## Conclusion

ITER-like monoblocks have been studied using a novel method in order to estimate the hydrogen content as a function of exposure conditions such as implanted particle flux, ion energy, heat load and monoblock surface temperature. Several hundred data points have been simulated with FESTIM and analysed to estimate the hydrogen inventory in monoblocks for any input conditions using Gaussian regression, a machine learning algorithm which calculates the confidence interval for each point, Thanks to this relation, one can easily estimate hydrogen content in the whole divertor without having to run all the simulations. An application has been made based on the output from a SOLPS calculation of exposure conditions distribution on the ITER divertor and shows that for these conditions the inventory could reach $10^{20}\,\hbox {H}$ per monoblock near strike points after a $10^{7}\,\hbox {s}$ exposure. The total hydrogen content in ITER divertor is estimated to be 8 g which is well below the inner-vessel safety limit of 1 kg.

Future work will include applying this technique to calculations performed on the WEST tokamak and studies of the impact of trap parameters (especially neutron-induced traps) on the inventory results as well as the impact of more complex geometries.

## Supplementary information

Supplementary Information
