# Supplementary material for: Parametric study of hydrogenic inventory in the ITER divertor based on machine learning
Source: Sci Rep. 2020 Oct 20;10:17798. doi: 10.1038/s41598-020-74844-w (PMC7576168; doi:10.1038/s41598-020-74844-w)
Supplement: Supplementary file 1 — Supplementary Information [file 41598_2020_74844_MOESM1_ESM.pdf]

Parametric study of hydrogenic inventory in the ITER divertor based on machine learning

Rémi Delaporte-Mathurin[1,2,*], Etienne Hodille[1], Jonathan Mougenot[2], Gregory De Temmerman[3], Yann Charles[2], and Christian Grisolia[1]

[1]CEA, IRFM, F-13108 Saint-Paul-lez-Durance, France
[2]Université Sorbonne Paris Nord, Laboratoire des Sciences des Procédés et des Matériaux, LSPM, CNRS, UPR 3407, F-93430, Villetaneuse, France
[3]ITER Organization, Route de Vinon sur Verdon, CS 90 046, 13067, St Paul Lez Durance Cedex, France
[*]corresponding author: remi.delaporte-mathurin@cea.fr

Appendix

Assuming a narrow Gaussian distribution for the source term φ_{imp}, the mobile particles concentration profile can be approximated by a triangular shape[1] (see Supplementary Figure 1).

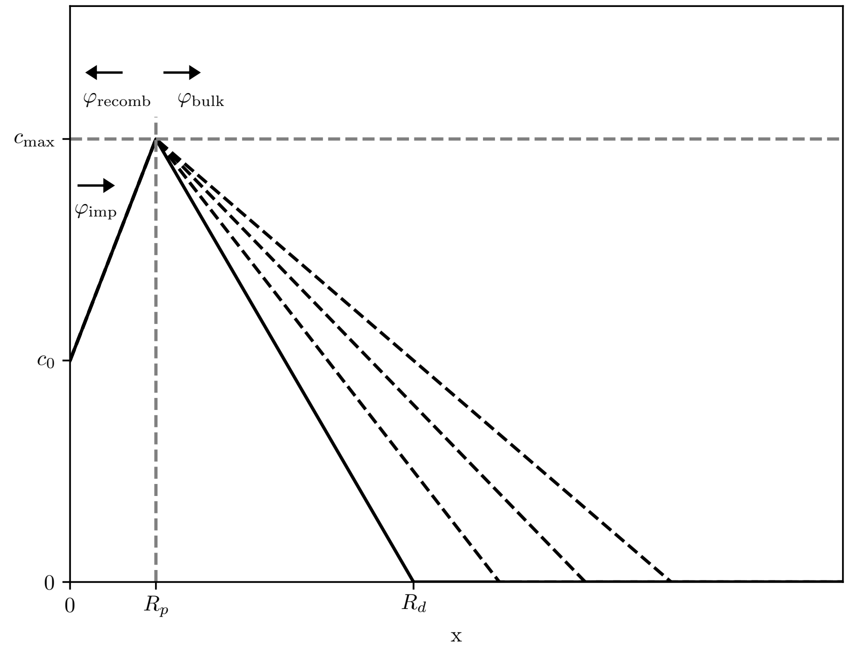

Figure 1. Concentration profile with recombination flux and volumetric source term at $x = R_p$. Dashed lines correspond to time evolution.

The expression of c_{max} can be obtained by expressing the flux balance at equilibrium:

$$\varphi_{\text{imp}} = -\varphi_{\text{recomb}} + \varphi_{\text{bulk}} \tag{1}$$

where $\varphi_{\text{recombination}}$ is the recombination flux and φ_{bulk} is the migration flux.

φ_{bulk} can be expressed as:

$$\varphi_{\text{bulk}} = D \cdot \frac{c_{\text{max}}}{R_d(t) - R_p} \tag{2}$$

When $t \rightarrow \infty$ or $R_d \gg R_p$ (a ratio of 10 or 100 is enough), $\varphi_{\text{bulk}} \ll \varphi_{\text{recomb}}$. According to Fick's law, Equation 1 reads:

$$\varphi_{\text{imp}} = D \cdot \frac{c_{\text{max}} - c_0}{R_p} \tag{3}$$

$$\Leftrightarrow c_{\text{max}} = \frac{\varphi_{\text{imp}} R_p}{D} + c_0 \tag{4}$$

Equation 1 can also be written by expressing $\varphi_{\text{recombination}}$ as a function of the recombination coefficient K:

$$\varphi_{\text{imp}} = K c_0^2 \tag{5}$$

$$\Leftrightarrow c_0 = \sqrt{\frac{\varphi_{\text{imp}}}{K}} \tag{6}$$

By replacing Equation 6 in Equation 4 one can obtain:

$$c_{\text{max}} = \frac{\varphi_{\text{imp}} R_p}{D} + \sqrt{\frac{\varphi_{\text{imp}}}{K}} \tag{7}$$

As the recombination process becomes fast (*ie* $K \rightarrow \infty$), $c_0 \approx 0$ and $c_{\text{max}} \approx \frac{\varphi_{\text{imp}} R_p}{D}$.

References

1. Schmid, K. Diffusion-trapping modelling of hydrogen recycling in tungsten under ELM-like heat loads. *Phys. Scripta* **T167**, 014025, DOI: 10.1088/0031-8949/T167/1/014025 (2016). Publisher: IOP Publishing.
